# Supplementary material for: The Role of the Basophil Activation Test in the Diagnosis of Drug-Induced Anaphylaxis
Source: Diagnostics (Basel). 2024 Sep 13;14(18):2036. doi: 10.3390/diagnostics14182036 (PMC11430944; doi:10.3390/diagnostics14182036)
Supplement: Supplementary file 1 [file diagnostics-14-02036-s001.zip › diagnostics-3179337-supplementary.pdf]

Name and surname of the examined
………

Age……
………..

Contact phone number and/or email address
……………………………………………………………………………………………………

Health information before vaccination against COVD-19

1. Do you suffer from chronic diseases?
   □ NO
   □ YES – what kind of?
   …….
   ……….
   ……….
2. Are you taking any medication?
   □ NO
   □ YES – which one?
   …….
   ……….
   ……….
3. Have you been diagnosed in the past with a severe, generalized allergic reaction (anaphylactic shock) after administration of medicine, food, or insect bite?
   □ NO
   □ YES – for what and when?
   …………………………………………………………………………………………………….
   ……………………………………………………………………………………………………….

4. Have you been diagnosed with an allergy to polyethylene glycol (PEG), polysorbate or other substances in the vaccine?
   □ NO
   □ YES – for what and when?……………………………………………………………………………………………….
   ……………………………………………………………………………………………………….
5. Have you been diagnosed with other allergies (inhalation allergens, food allergens, latex, insect venoms, medications)?
   □ NO
   □ YES – for what and when?
   …………………………………………………………………………………………………….
   ……………………………………………………………………………………………………….

COVID-19 vaccination information

1. Date and time of administration of the 1st dose of the ……………………………………………………………………………… vaccine
2. Date and time of the 2nd dose of the vaccine (*if applicable) …………………………………………………….

3. Vaccine name
   ....
4. Date and time of onset of adverse reactions after vaccination ....
   ....

   a) Have you experienced symptoms involving the skin?
      □ NO
      □ YES - itching of the skin (localized - > describe the location or generalized?) ....
      ....
      □ YES - urticaria (local - > describe location or generalized?) ....
      ....
      □ YES - other (what?, in what location?) ....
      ....

   b) Have you experienced symptoms involving the respiratory system?
      □ NO
      □ YES - (what?)
      ....
      ....

   c) Have you experienced abdominal symptoms?
      □ NO
      □ YES - (what?)
      ....
      ....

   d) Have you experienced cardiovascular symptoms? What was your blood pressure and heart rate?
      □ NO
      □ YES - (what?)
      ....
      ....
      Blood pressure measurement:
      ....
      Heart rate measurement:
      ....

   e) Has there been a loss of consciousness?
      □ NO
      □ YES

   f) Have you experienced other symptoms?
      □ NO
      □ YES - (what?)
      ....
      ....
      ....

5. Was it necessary to visit the ambulance / emergency room / hospitalization?
   □ NO
   □ YES - (describe the type and length of medical intervention)
   ....

…….

6. What treatment was given?
 □ none
 □ oxygen therapy
 □ fluids intravenously
 □ adrenaline intramuscularly
 □ antihistamines
 □ glucocorticoids
 □ other (what?)
 ……….
